# Transactions of the Antiseptic Club

**Published:** 1895-08

**Authors:** 


### TRANSACTIONS OF THE ANTISEPTIC CLUB. Reported by Albert Abrams, a member of the San Francisco medical profession. Illustrated. New York: E. B. Treat, 5 Cooper Union. 1895. Price, $1.75.

This unique book is a caricature of the present craze for antisepsis. It is a story of the doings of a lot of doctors who are strong advocates of the germ theory of disease, and, of course, all their acts are an unmerciful satire of the whole science of bacteriology.

The author is a clever wit, and hence his book is highly entertaining, and must prove a great success.

